# Catalyst shape engineering for anisotropic cross-sectioned nanowire growth

**DOI:** 10.1038/srep40891

**Published:** 2017-01-20

**Authors:** Yonatan Calahorra, Alexander Kelrich, Shimon Cohen, Dan Ritter

**Affiliations:** 1 Department of Electrical Engineering, Technion - Israel Institute of Technology, Haifa 32000, Israel

## Abstract

The ability to engineer material properties at the nanoscale is a crucial prerequisite for nanotechnology. Hereunder, we suggest and demonstrate a novel approach to realize non-hemispherically shaped nanowire catalysts, subsequently used to grow InP nanowires with a cross section anisotropy ratio of up to 1:1.8. Gold was deposited inside high aspect ratio nanotrenches in a 5 nm thick SiN_x_ selective area mask; inside the growth chamber, upon heating to 455 °C, the thin gold stripes agglomerated, resulting in an ellipsoidal dome (hemiellipsoid). The initial shape of the catalyst was preserved during growth to realize asymmetrically cross-sectioned nanowires. Moreover, the crystalline nature of the nanowire side facets was found to depend on the nano-trench orientation atop the substrate, resulting in hexagonal or octagonal cross-sections when the nano-trenches are aligned or misaligned with the [1̄10] orientation atop a [111]B substrate. These results establish the role of catalyst shape as a unique tool to engineer nanowire growth, potentially allowing further control over its physical properties.

For the purpose of controlling and improving semiconducting nanowire (NW) devices for various applications^1^,^2^,^3^,^4^,^5^,^6^,^7^, an extensive research effort has been invested in studying NW growth; this was typically achieved by studying the different effects of user controlled parameters: (i) materials - including type and size of NW catalyst, the growth substrates and precursors, and (ii) by altering growth-system parameters, i.e., temperature and precursor flow^8^,^9^,^10^,^11^,^12^,^13^,^14^,^15^,^16^,^17^. In the following report we present a new paradigm, that of catalyst shape engineering, as a useful tool to control NW growth results; in particular, we show that by imposing a non-hemispherical shape to the catalyst-substrate interface, anisotropic cross-sectioned NWs may be grown - NWs with potentially new physical characteristics. Furthermore, we show that the combination of a non-hemispherical catalyst with different crystalline orientations of the long axis results in non-trivial side-faceting of the grown NWs.

Nanowire cross section shape and faceting is expected to influence its electrical, mechanical, optical and chemical properties^18^,^19^,^20^,^21^,^22^,^23^,^24^. In particular, Foster *et al*. have shown that the emission of InGaAs quantum wells embedded inside selective-area-grown anisotropically cross-sectioned GaAs NWs, exhibited linear polarization aligned with the long cross-section axis; basically, when one cross-sectional length-scale is too small to support an optical mode, the spatial degeneracy is lifted and the photoluminescence becomes linearly polarized^22^. This work is an excellent reference for catalyst free growth of anisotropic cross-sectioned NWs, and will be discussed below in further detail. Similar results have been reported by Li *et al*., in regards to polarisation of laser emission from top-down fabricated GaN NWs^23^. In a different case, Mankin *et al*. have studied the reactivity of different facets of a VLS grown SiNW; over-grown CdS shells were found to selectively form on {110} and {111} facets, while {113} facets remained bare. They suggest that differences in the nature of the native oxide grown on the various facets results in an efficient passivation on {113} facets^24^. These examples demonstrate how control and understanding of surface shape and facet orientation open up further possibilities to engineer other physical properties. The only report we are aware of, trying to link NW catalyst shape with its properties, is the report by Lin *et al*., where round and faceted (shaped) gold nanoparticles were used to catalyze InAs NWs. Their results show faceted catalysts induced a higher growth rate to the NWs, however no other distinctions were found^25^.

The underlying concept which allows efficient manipulation of the catalyst shape in this work, is growth by the selective area vapor-liquid-solid (SAVLS) mechanism - where the metal catalyst is deposited within an opening in a selective growth mask (usually SiO_2_ or SiN_x_); see refs 26, 27 for a comprehensive overview of this growth regime. When a metallic layer is deposited on top of a mask-less substrate and subsequently heated, a process of agglomeration through dewetting is expected to change the layer morphology^28^,^29^; in regards to mask-less NW growth, this property is used to rapidly realize NW catalysts - following the deposition of a thin layer of metal^30^,^31^,^32^.

Recently, we have studied the effect of a selective area mask on the dewetting of large catalyst discs (100–450 nm in diameter). It was found that the mask edges prevent the migration of catalyst droplets outside the opening area, and increase the chances of agglomeration into a single particle; as a result, up to 3 fold reduction of the NW diameter compared to the original lithography was obtained simply by using the SAVLS approach. Moreover, it was found that a final shape of a hemisphere adequately describes the catalyst agglomeration^33^.

We suggest utilizing these findings is useful to realize anisotropic catalysts: consider a metal stripe of width *W*_*M*_, thickness *T* and length *L* deposited in a trench of a slightly larger width of *W*; the total deposited volume is therefore *V*_*M*_ = *W*_*M*_*TL*. The diameter of a hypothetical equilibrium hemisphere resulting from this metal stripe is given by


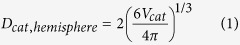


where *V*_*cat*_ is the effective volume, increased due to incorporation of substrate material inside the metal at a volumetric fraction *β*, (e.g., silicon, germanium, or group III elements), sustaining *V*_*cat*_ = *V*_*M*_/(1 − *β*). It stands to reason that in case the equilibrium catalyst diameter is larger than the trench width, i.e. *D*_*cat*_ > *W*, a non-hemispherical shape for the catalyst-substrate interface is expected - under the assumption that the catalyst does not wet the mask edges efficiently, as has been demonstrated in our previous study^33^. The suggested process is shown schematically in Fig. 1a, depicting side- and top-view of the metal stripe evolution, from deposition to NW growth. Figures 1b,c show a schematics and a corresponding SEM image of the growth site: three orientations of metal stripes inside lithography defined openings in a selective area mask, atop the InP growth wafer (see experimental section below for further details). A detailed study regarding controlled dewetting of gold on trench-patterned substrates was recently published by Lu *et al*., demonstrating a high level of control over the resulting nano-patterns^34^.

## Results and Discussion

The following account of the results is mainly focused on two aspects, comprising the core of this work: first, the ability to induce anisotropic agglomeration of the gold within the nanotrenches, and the subsequent growth of anisotropically cross-sectioned NWs; second, the effect of nanotrench orientation on the faceting of grown NWs. In addition, findings regarding the unintentional growth of non-vertical square cross-sectioned NWs will be presented. For consistency, the SEM results in Figs 2, 3 and 4 are presented such that the 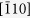 is pointing to the right.

### Anisotropically cross-sectioned nanowires

Figure 2 shows growth results from individual ~90 nm nanotrenches, variously oriented, with 12 nm of deposited gold (see supporting information for array view). Figures 2a–c correspond to nanotrenches at 45°, 30° (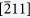) and 0° (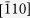) rotation; it can be seen that similar sizes of the catalyst were obtained on the different locations and that indeed, anisotropically cross-sectioned NWs may be grown in the proposed method. The NWs shown in Fig. 2a–d are relatively short, about 350 nm, due to the large catalyst inducing a low growth rate (blue rectangle); the results of a prolonged growth are shown in Fig. 2e–g. Figure 2d shows a nanotrench similar to the one depicted in Fig. 2c, with the distinction that the catalyst did not maintain the confinement; consequently, the anisotropic nature of the catalyst was lost, as evident by the width projection measured in the 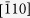 direction which is about 130 nm, compared to about 180 nm in Fig. 2c. Furthermore, in order to examine the ability to grow longer NWs maintaining the confinement, an extended growth was performed, which consisted of 10 minutes at 420 °C followed by 50 minutes at 480 °C (this growth is marked by a red rectangle throughout the paper); the results are shown in Fig. 2e–g. Remarkably, the NW catalysts maintained the confinement of the nanotrench to grow *μ*m scale NWs. Figure 2g_2_ shows the top view of the NW depicted in Fig. 2g_1_, establishing that indeed an anisotropic cross-section has been obtained throughout the NW length. Some roughening of the sidewalls is observed following the transition to 480 °C, which might be related to an increased density of stacking faults related to a higher growth rate; however, these results prove the viability of the suggested process as a route to realizing high aspect ratio NWs with an anisotropic cross section.

The problem of template controlled dewetting has been considered be several authors^34^,^35^. In addition to the already complex nature of this phenomenon, our work introduces two significant non-idealities: (i) the different nature of the mask and substrate, where different interface energies dictate the preferred wetting of the substrate^33^; (ii) significant interaction between the substrate and the catalyst, deviating from pure dewetting^34^. Nonetheless, the ideal model recently presented in ref. 34, still points out to the critical role of dimension interplay between layer thickness, trench width and trench depth in the dewetting process. The tolerance of the method was checked by deposition of thinner and thicker metallizations (6 and 18 nm; see supporting information). It was found that the thin metallization resulted in non-agglomerated nanoparticles, essentially inducing the growth of commonly encountered NWs. Conversely, the thick metallization resulted in an increased tendency of the catalyst to lose confinement by the mask edges, allowing the catalysts to spill outside the opening (similar to Fig. 2d). Nonetheless, when the confinement was not lost, higher anisotropic aspect ratios were obtained per a given nanotrench length compared to 12 nm metallization - as would be expected due to the increased amount of gold. The obtained results indicate that the scalability of this method requires optimization.

### Nanowire side facet engineering

We now turn to take a closer look at the growth results, with particular consideration of growth from different trench orientations. Figure 3a–c shows a high magnification, top view, of typical growth results (when catalysts are successfully confined) from ~80 nm wide, 1 *μ*m long, nanotrenches at 45°, 30° (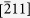) and 0° (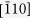) rotation; the deposition thickness was 12 nm. The top point of view allows careful examination of the catalyst and NW circumference; indeed, the anisotropic nature of the cross-section is evident. These NWs exhibit an anisotropic ratio of about 1:1.5, with the NW cross section short axis measuring at about 100 nm - somewhat larger than the trench width (marked by yellow dashed lines). This could be brought by “spillage” of the catalyst when the growth front protrudes out of the selective area mask, or by a certain degree of radial growth. By plugging-in the process dimensions indicated above (We account for a 30 nm wide deposition, with the rest of the trench resulting from wet-etch broadening) and assuming 45% of indium in the catalyst^36^, into Eq. 1, an equilibrium hemispherical catalyst diameter of 135 nm is calculated - indicating that indeed, confinement is expected since the trench width is 80 nm. Lower aspect ratios were observed; for example, 1:1.65 when the deposition is thicker (18 nm) for similar 1 *μ*m lithography (see Supporting Information - Fig. S2e), down to 1:1.8 (110:200 nm), which is the lowest ratio observed in this set of experiments, for a 2 *μ*m long nanotrench exhibiting single particle agglomeration in the conditions mentioned above (see Supporting Information - Fig. S3).

Furthermore, an important finding is that the misoriented trenches induced an octagonal circumference to the NWs, as can be seen in Fig. 3a,b. This stands in contrast compared to a nearly perfect hexagonal cross-section in the 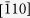 trench orientation (“standard” hexagonal faceting is also evident in Figs 2g_2_ and 3d). The inset in Fig. 3 schematically shows the 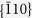 and 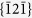 Zinc-blende (ZB) facet families and their corresponding Wurtzite (WZ) counterparts: 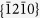 and 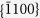; thus allowing the understanding of the grown NWs facet configurations. If so, 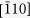 oriented trenches induced the growth of hexagonal NWs with 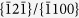 type facets (marked red), while the octagonal NWs, grown from misoriented trenches, have six 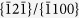 facets, with an addition of two 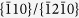 facets (marked blue). This result already shows that the NWs grown from nanotrenches aligned with different crystalline orientations, may posses different physical properties due to the two additional facets of a different family. Fonseka *et al*. have reported the growth of [100] oriented InP NWs with octagonal cross-sections in the NW section close to the catalyst; in that case, lateral growth renders a square cross-section to the remainder of the NW, and the details of the growth as a whole are controlled by standard growth conditions - temperature and precursor flow^37^. This picture stands in contrast to the micron-scale anisotropic cross sections demonstrated in Fig. 2e–g.

In order to understand the growth of octagonal cross-sectioned NWs, we first examine the growth of the hexagonal cross-sectioned NWs. Interestingly, non-confined NWs also exhibit hexagonal 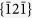 faceting; as shown in Fig. 3d, where a catalyst stripe did not yield a single particle, and the smaller residues resulted in the growth of symmetrically cross-sectioned NWs with visible 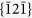 facets. Sibirev *et al*. have calculated different facet-associated surface energies of III-V NWs for WZ and ZB configurations; their calculations exhibited a high degree of agreement with reported experimental results^38^. Table 1 shows the calculated surface energies of 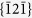 and 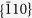 facets for InP and GaAs. Although the calculations are based on a simplified nearest neighbor model, a useful rule of thumb can be outlined: for WZ nanowires, grown along the 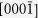/[111]B directions, the 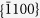 (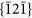) faceting is energetically favored; conversely, for ZB nanowires grown along the same axis, the 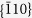 (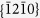) is preferred (this has also been demonstrated by Ikejiri *et al*.^39^). For the growth system used in this study, under a relatively wide range of conditions, WZ structure is most commonly observed, exhibiting high purity levels, up to the absence of stacking faults^16^. Indeed, the unconfined NWs in Fig. 3d exhibit the preferred 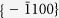, facets in agreement with the theoretical predictions.

**Figure 1:**
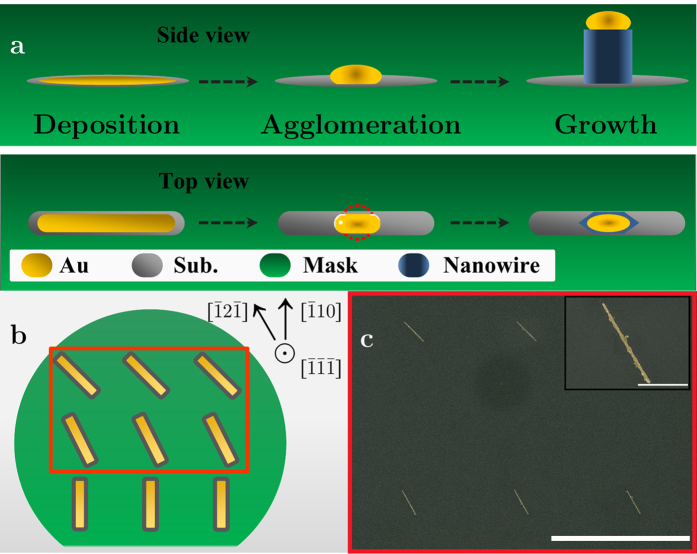
Catalyst shape engineering. (**a**) Side- and top-view schematics of the suggested route to realize anisotropic cross-sectioned catalysts and NWs: trench-like openings in a selective area mask, induce anisotropic agglomeration of the catalyst to form anisotropic NWs. The dashed circle indicates the equilibrium shape, not reached during this process. (**b**) Top-view schematics of the nanotrench orientations, with trenches aligned at 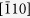, 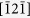 and 45° with respect to 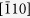. The wafer [110] flat was used for lithography orientation. (**c**) False-colored SEM images of 1 *μ*m nanotrenches with 12 nm of gold deposited within; the red rectangle in [b] schematically depicts this area. The inset shows a close-up on one of the nanotrenches oriented along the 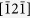 direction. Scale bars are 5 *μ*m and 500 nm for the inset.

**Figure 2:**
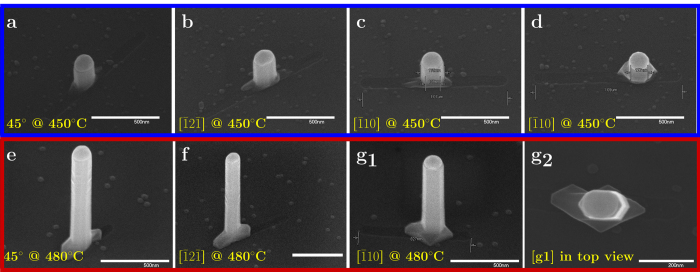
Anisotropic cross-sectioned NWs. Scanning electron micrographs (all but [**g2**] are 30° tilted) of growth results from ~90 nm nanotrenches with 12 nm of deposited gold: (**a**–**d**) growth from different orientations of 1 *μ*m nanotrenches at 450 °C; (**e**–**g**) an extended growth, where an additional stage at 480 °C was performed - leading to further axial growth. [**g2**] shows the same NW as [**g1**]; [**a–g**] scale bars are 500 nm, [**g2**] is 200 nm.

Next, it is beneficial to examine the catalyst-free anisotropically cross-sectioned GaAs NWs reported by Foster *et al*., under these theoretical guidelines^22^. To realize such NWs, they have created asymmetric trench-like openings in a selective area growth mask; the trenches were aligned with various 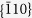 and 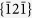 type orientations. Notably, their growth is purely based on the selective area approach and is catalyst-free. Their main experimental observation was that 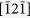 oriented trenches resulted in the growth of anisotropically cross-sectioned hexagonal NWs, having 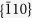 facets; while 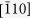 oriented trenches yielded more symmetrical hexagonal structures, having the same 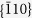 facets (see ref. 22 Figs 1 and 2). Although not directly stated in that publication, previous publications from that group regarding GaAs NWs in similar conditions, indicate these NWs are ZB with 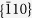 Faceting^40^,^41^. Once again, the crystalline structure and side-facets are in good agreement with the surface energy prediction by Sibirev and co-workers.

**Figure 3:**
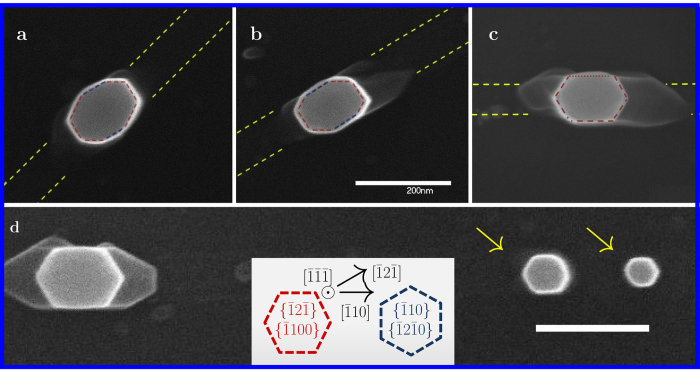
Crystalline orientation dependence of NW cross-section. Top view scanning electron micrographs of typical growth results from (**a**,**b**,**c**) nanotrenches of ~80 nm width and 1 *μ*m length with 12 nm of deposited gold; [a] −45°, [b] −30° and [c] −0° rotated relative to 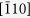 orientation. Red and blue dashed lines mark the top-viewed facets of the NW, and yellow dashed lines mark the nanotrench. (**d**) Nanotrench of ~80 nm width and 2 *μ*m length with 12 nm of deposited gold in the 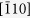 orientation; the agglomeration is not perfect and arrows mark two catalyst particles which resulted in growth of symmetrical NWs. Inset shows schematic of the facet orientations in ZB and WZ notations. Blue dashes represent the 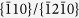 family, and red dashes the 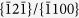 family of facets.

We can now point out the differences and similarities between the catalyst-assisted and catalyst-free growth of NWs. In both cases, when the nanotrench orientation is such that facets normal to the long axis are energetically favored, hexagonal anisotropically cross-sectioned NWs are grown, maintaining the preferred faceting (for either WZ or ZB). A crucial distinction between the methods is observed when the nanotrenches are oriented such that the long-axis normal facet is not compatible with the energetic criteria (as in Fig. 3a,b above and Fig. 2 in ref. 22): for the catalyst-free case, the energy considerations still dictate the growth, resulting in a smaller extent of asymmetry in the cross-section; thus limiting the ability to grow such structures in orientations other than the energetically preferred ones. Contrary, in catalyst assisted growth, the catalysts, and subsequently the NWs, maintain confinement *despite* the facet energy considerations, and the asymmetrical shape of the droplet. The confinement and the energy considerations are reconciled by the additional facets, leading to the octagonal cross-section observed - effectively opening up new possibilities to engineer NW properties. Importantly, the confinement is maintained not only in “natural” nanotrench orientations such as 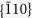 and 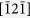, but also in the 45° oriented nanotrenches, which in terms of crystalline symmetry could be considered “arbitrary”.

The maintained confinement, which resulted in the growth of increased energy facets, was observed on-top of the already less energetically favored non-hemispherical catalyst; apparently, both the facet and droplet energetic considerations make this observation improbable. We examine this result from a phenomenological point of view, in order to obtain better insight into the growth of octagonal NWs. Consider a 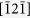 oriented nanotrench, as seen in Fig. 3b. Prior to growth, due to surface (energy) minimization, the agglomeration results in a droplet which is as close as possible to a hemisphere, after which growth commences. From that point, there are two routes for a hexagonal NW to be grown (see Supporting Information - Fig. S4): (1) loss of confinement, with a hemispherical-like catalyst (similar to growth from non energetically favored trenches in the report by Foster *et al*. in ref. 22); (2) decrease of interfacial area, accompanied by increase of the catalyst/ambient interfacial area, in a symmetrical or asymmetrical manner, such that the catalyst/NW interfacial shape is aligned with the energetically favored facets.

Consider the first option: for the catalyst to become more hemispherical, it needs to wet either the NW edges, or the mask (in the initial growth stages); both processes come at an energetic cost^29^. Indeed no actual growth of vertical NWs was observed in the cases where confinement was lost. The implications of the second option are that the total surface area of the catalyst is increased (the cost of a lower interfacial area), and with it its energy, which is also unfavorable. Therefore, based on this qualitative analysis, we conclude that although the initial state considered seems improbable, it is favored compared to the alternatives for lowering the facet energies, given that the NW has already started growing these facets. The octagonal NWs could be viewed as growing in a metastable state, reconciling the energetic demands of the catalyst and the NW facets. This observation could be an extension of the geometrical frustration concept for ZB NWs^42^.

### Growth of square cross-sectioned nanowires

**Figure 4:**
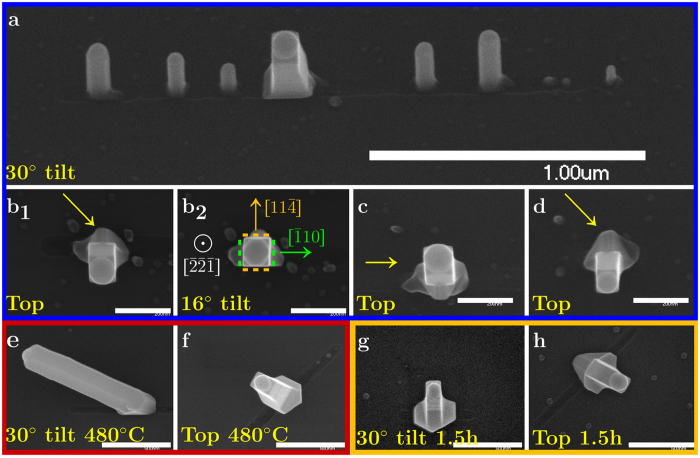
Growth of square cross-sectioned NWs. Scanning electron micrographs of NWs with square/rectangular cross section; [**a,b_2_,e,g**] are tilted-view while others are top-view. (**a**–**d**) Results from a 450 °C growth (blue rectangle - same growth as in Fig. 2a–d). The sample was tilted until the NW in [**b**_**1**_] reached a vertical point of view at 16° tilt - [**b**_**2**_]. The arrows point to tetrahedron-like structures found at the base of cubic NWs, and calculated facet orientations are shown in [**b**_**2**_]; (**e**,**f**) results from a 480 °C growth (red rectangle - same growth as in Fig. 2e–g); (**g**,**h**) Results from a 90 minute 450 °C growth (yellow rectangle). Scale bars are 1 *μ*m [**a**], 200 nm [**b–d**] and 500 nm [**e–h**].

Finally, we report the growth of NWs with square and rectangular cross-sections; a rare finding on (111)B III-V substrates. This consistently occurred in about 2–4% of the growth locations where confinement was lost and a round catalyst was seen at the top of an epitaxially grown NW. Figure 4 shows several examples of such NWs, grown at various conditions, observed in top view and tilted view. Figure 4a shows an example where growth from a 2 *μ*m nanotrench, without successful single-particle agglomeration, resulted in the growth of a square cross-sectioned NW among other 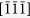 NWs; this is an image taken at 30° tilt, nonetheless it already demonstrates the “off-axis” nature of these NWs, as subsequently seen in the top-view images.

The most common occurrence of these NWs was in the 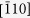 oriented nanotrenches, while in the 45° orientations, fewer such NWs were observed (Fig. 4f,h); interestingly, in the 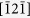 orientations no such NWs were found - an issue for future research, together with the general growth mechanism of these NWs. When different growth conditions were used (differently colored frames), it was found that these NWs are able to elongate along the growth direction (alongside radial growth, as indicated by the difference between catalyst and NW diameters in Fig. 4e), and that in prolonged growth times a rectangular cross section is developed - indicating different growth rate of side-facet pairs (Fig. 4f–h).

The full analysis of the NWs’ growth mechanism and structure is a subject for future work, however, a preliminary analysis, based on SEM directional examination, was performed for the most commonly observed NWs (such as those seen in Fig. 4b–d). While keeping the NW in Fig. 4b_1_ in view, the sample was tilted until the side facets were not visible, indicating an aligned top-view; this occurred at 16° tilt. In addition, this NW is obviously orthogonal to the nanotrench, therefore two of its side facets are 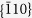 type. The orientation exhibiting 16° and 90° with 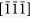 and 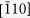 (correspondingly), is 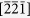 (accurately - 15.79° with 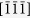); the two other side facets in this case are 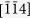 and 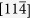. This makes a remarkable finding, considering that reports of square/rectangular NWs usually regard the [110]/[100] growth orientations^37^,^43^,^44^.

Interestingly, for NWs pointing to the other way (e.g. Fig. 4c) top-view alignment was found in 16° again, when the “mirrored” procedure was performed. It is worth examining the base of the NWs in Fig. 4b,d compared to Fig. 4c (marked by arrows): the top view reveals a mirror symmetry of the tetrahedron base, relative to the 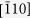 orientation. In a publication by Ikejiri *et al*., similar structures were found during growth of selective area GaAs NWs; it was suggested that twining is responsible for the mirror-like inversion of the tetrahedron orientation^45^. If so, it is probable that a twin defect results in the symmetry observed here for the growth of square cross-sectioned NWs, atop mirrored tetrahedrons, at both 16° and −16° relative to the substrate normal, within the 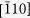 plane.

The square cross section of these NWs was found to develop into a rectangular cross section in the prolonged 90 minute growth (yellow frame). This kind of NW evolution is usually attributed to different growth rate of different facets; in particular, Shtrikman *et al*. have demonstrated rectangular NWs having 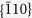 and {100} facets (opposite two of each type), with a slow growth rate of the 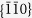 facets^43^. In our case, as the 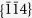 type facets are composed of micro {100} and {111} facets^46^,^47^, it is similarly expected they have a higher growth rate compared to the 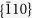 facets - as indeed observed (Fig. 4g,h - notice that the tetrahedron in Fig. 4h is mirrored). Further research, focused on TEM examination of these NWs is necessary to fully understand their growth, and to verify their growth direction.

## Conclusion

We presented a method to grow anisotropically cross-sectioned catalyst assisted NWs. At the heart of this method lies the ability to engineer the shape of the catalyst, and the catalyst/substrate interface. We observed NWs with an anisotropic cross-section ratio of up to 1:1.8, and have demonstrated the ability to grow such NWs to *μ*m-scale length. The orientation of the long asymmetry axis atop the growth substrate was found to have a profound effect on growth, where nanotrenches aligned (misaligned) with the 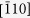 orientation resulted in NWs of hexagonal (octagonal) cross sections; the two additional facets are of different crystalline orientation, compared to the six “energetically trivial” facets, and may induce different chemical properties to the octagonal NWs. In addition, a consistent fraction of the growth sites resulted in growth of NWs having square/rectangular cross sections, a remarkable finding in growth on (111)b III-V substrates. Preliminary directional analysis of these NWs suggests that most of them grew in the 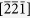 direction, with 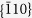 and 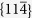 type facets.

This report presents a new paradigm in bottom-up catalyzed growth of NWs, demonstrating that the catalyst shape prior to growth can be controlled inside the growth chamber, and subsequently impose a spatially non-symmetric growth interface. Furthermore, the significance of the catalyst exceeds the mere shaping of NW growth interface; as indicated by growth of octagonal NWs, and off-axis cubic NWs. These results open-up a variety of future research directions, from devices based on anisotropically cross-sectioned NWs and NW facet engineering, to fundamental studies regarding the catalyst-mask-substrate interaction and the growth of cubic NWs on (111)B substrates.

## Methods

InP Nanowires were grown on (111)B oriented InP substrates (Semiconductor wafer Inc.) by metal organic molecular beam epitaxy (MOMBE). The sample was first coated by ~5 nm of SiN_x_ by PECVD to act as a selective area mask for the growth. Subsequently, the sample was spin-coated with PMMA (495 A5 Microchem), and line patterns with lengths ranging from 300 nm to 2 *μ*m and widths of about 30–60 ±5 nm were difined by e-beam lithography; most of these lines were aligned with the 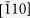 direction, however 1 *μ*m lines were written also at 30° and 45° relative to that orientation - the former corresponds to 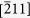 (see manuscript Fig. 1b). Directional alignment was performed relative to the wafer flat. Using this lithography pattern, the mask was etched by buffered HF (resulting is a 20–30 nm widening of the nanotrenches), and gold (6, 12 or 18 nm) was deposited within the etched nanotrenches. Since wet etching is used to open-up the nanotrenches, a widening of their width compared to the original lithography is unavoidable. An etching step with better directionality (e.g., RIE) would be more efficient in controlling the aspect ratios of the resulting catalyst; a similar process was presented by Foster and co workers^22^. The samples were inserted into the growth chamber and heated to 455 °C under PH_3_ flux prior to growth - performed at 450 °C for 30 minutes (blue rectangles throughout the paper), unless otherwise stated.

Scanning electron microscopy (Hitachi S-4700) with magnification of up to 200 k was used to characterize the growth results, in terms of dimensions, orientations and faceting.

## Additional Information

**How to cite this article**: Calahorra, Y. *et al*. Catalyst shape engineering for anisotropic cross-sectioned nanowire growth. *Sci. Rep.* **7**, 40891; doi: 10.1038/srep40891 (2017).

**Publisher's note:** Springer Nature remains neutral with regard to jurisdictional claims in published maps and institutional affiliations.

## Supplementary Material

Supplementary Information

## Figures and Tables

**Table 1:** WZ and ZB associated surface energies for InP and GaAS^38^.

Material	Facet Pair	WZ Energy [J/m^2^]	ZB Energy [J/m^2^]
InP	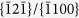	1.19	1.56
	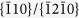	1.38	1.38
GaAs	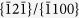	1.3	1.79
	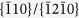	1.54	1.54
