# Supplementary material for: Catalyst shape engineering for anisotropic cross-sectioned nanowire growth
Source: Sci Rep. 2017 Jan 20;7:40891. doi: 10.1038/srep40891 (PMC5247733; doi:10.1038/srep40891)
Supplement: Supplementary Information [file srep40891-s1.pdf]

Catalyst shape engineering for anisotropic cross-sectioned nanowire growth
Supporting Information

Yonatan Calahorra, Alexander Kelrich, Shimon Cohen, Dan Ritter

Figure S1 shows a part of growth array, with 45° rotated nanotrenches. The single particle yield is 80%, which was characteristic for this array. A closer look reveals not all catalyst result in the growth of NWs.

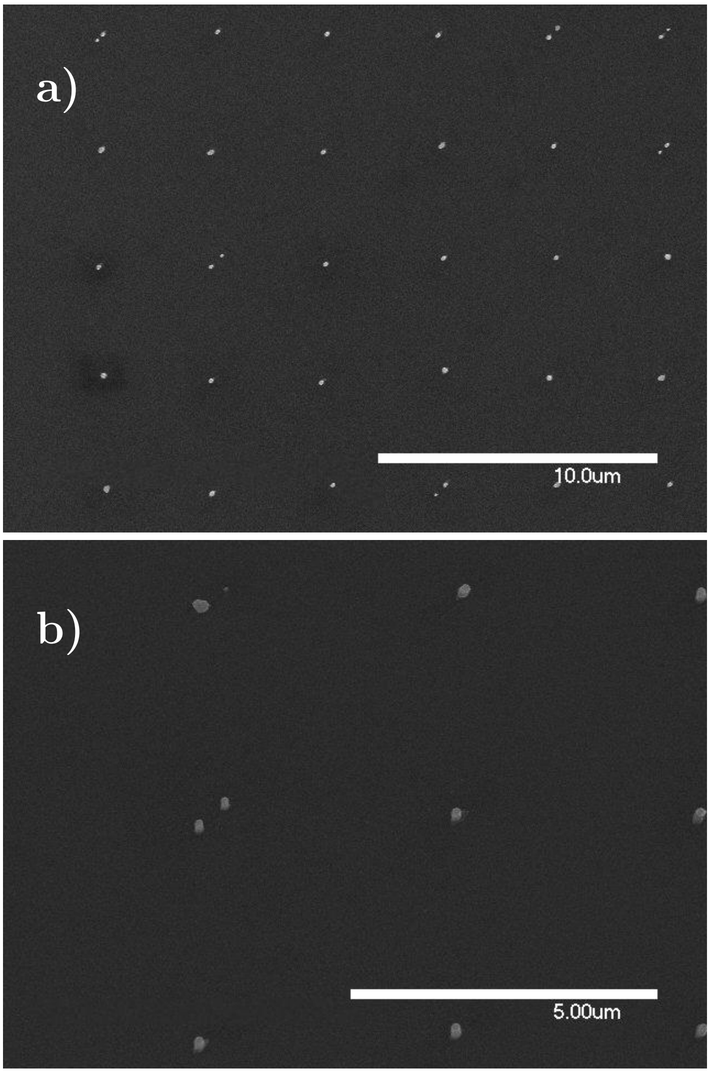

Figure S1: a) Tilted SEM image of 45° rotated nanotrenches; b) The same location at higher magnification.

Figure S2 Shows growth from samples of 6 and 18 nm thick gold metallization. Compared to the 12 nm metallization (main text), the single particle yield is lower for the 6 nm deposition, and the degree of catalyst confinement is reduced as well - due the smaller volume of the catalyst (Fig. S2b). On the other hand, in 18 nm metallization, while an increased tendency of the catalyst to loose the confinement was observed (Fig. S2d,f compared to Fig. S2c,e), lower asymmetry ratios were achieved when the confinement was maintained.

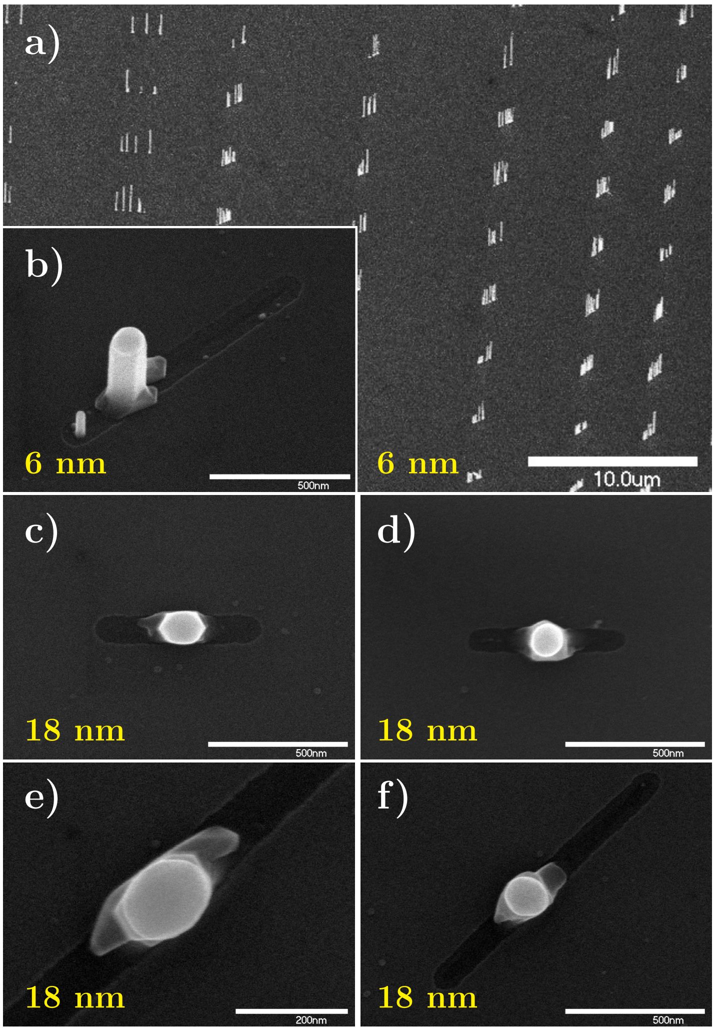

Figure S2: a) Tilted SEM image of 30 45° rotated nanotrenches; b) The same location at higher magnification.

Figure S3 shows a top view of the highest asymmetry ratio observed - 1:1.8 (110:200 nm); this was found on a 2μ nanotrench, with successful single particle agglomeration. As with other NWs seen in this work, there is some broadening of the short axis compared to the width of the opening (about 80 nm in this case).

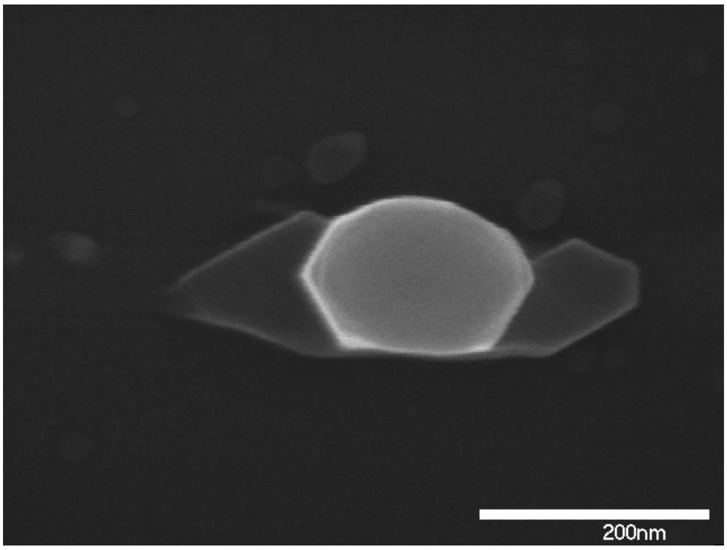

Figure S3: SEM image of a 2μ nanotrench growth site with a single catalyst particle of 1:1.8 asymmetry ratio.

Figure S4 schematically describes the routes by which the catalyst atop an octagonal NW could transform into a catalyst growing a hexagonal NW.

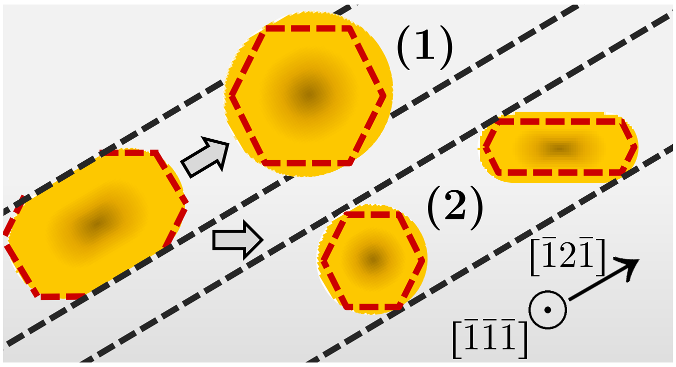

Figure S4: Schematic top-view illustration of the two hypothetical destabilization routes for the octagonal NW catalyst, and the subsequent growth of an hexagonal NW having only low energy facets. (1) and (2) designate the two routes mentioned in the main text.
